# Presumed Congenital Urethrorectal Fistula with Suspected Distal Urethral Atresia in a Kitten Managed with Modified Perineal Urethrostomy: A Case Report

**DOI:** 10.3390/vetsci13101027

**Published:** 2026-09-29

**Authors:** Guiqun Niu, Guoyang Sun, Chenyiyu Zheng, Shuai Li

**Affiliations:** 1 Shanghai Veterinary Research Institute, Chinese Academy of Agricultural Sciences, Shanghai 200241, China; 2 Shanghai Linghua Pet Hospital Co., Ltd., Shanghai 201100, China; niuguiqun@163.com; 3 Shanghai GlinX Biotechnology Co., Ltd., Shanghai 200135, China; guoyang9898@outlook.com (G.S.); longyu_3_yulong@163.com (C.Z.)

**Keywords:** cat, congenital anomaly, perineal urethrostomy, urethral atresia, urinary diversion, urethrorectal fistula

## Abstract

Congenital urethrorectal fistula is exceptionally uncommon in cats and may be difficult to anatomically define when accompanied by distal urethral maldevelopment. A 4-week-old intact male domestic shorthair kitten weighing 745 g was evaluated for watery feces, persistent perianal wetness, and suspected passage of urine through the anus. Manual bladder compression produced dark anal discharge with a creatinine concentration of 1202 µmol/L. Ultrasonography showed a distended, thick-walled bladder and proximal urethral dilation. CT retrograde urethrography demonstrated contrast within the penile urethra, marked dilation of the pelvic urethra, and a blind diverticulum-like pouch, although the fistulous tract was not visualized. The findings supported a presumptive diagnosis of congenital urethrorectal fistula with distal urethral atresia or discontinuity. A modified perineal urethrostomy was performed by anastomosing preserved preputial mucosa to the proximal urethra, establishing urinary diversion without exploring or closing the tract. Urinary outflow and fecal consistency improved, and the upper urinary tract was ultrasonographically unremarkable at postoperative day 25. Stomal stenosis prevented normal urination at day 675 and necessitated a second surgery. At day 995, urination was generally unobstructed, although intermittent urinary leakage and hematuria persisted. In conclusion, combined fluid analysis and multimodal imaging can support recognition of congenital urinary–rectal communication in kittens. Modified perineal urethrostomy can provide urinary diversion when normal outflow cannot be restored, but long-term surveillance is required because leakage and stomal stenosis may occur.

## Simple Summary

A rare birth defect can create an abnormal connection between the tube that carries urine from the bladder and the lower bowel. This may cause urine to pass through the anus, leading to watery stools, constant wetness around the anus, infection, and difficulty urinating. We report the diagnosis and treatment of a four-week-old male kitten with these signs and severe abnormal development of the lower urinary tract. Testing of the fluid discharged from the anus showed a high creatinine concentration, supporting the presence of urine. Ultrasound and computed tomography showed marked enlargement of the bladder and abnormal dilation and interruption of the urethra, although the exact abnormal connection could not be directly identified. Surgery was therefore performed to create a new opening for urine using the kitten’s own preputial tissue, without directly repairing the connection. After surgery, the kitten urinated through the new opening, passed normal stools, and gained weight, although intermittent urine leakage persisted. Stomal stenosis later prevented normal urination and required a second operation; by postoperative day 995, urination was generally unobstructed and urinary control had improved, although intermittent leakage and hematuria remained. This case may help veterinarians recognize similar congenital abnormalities and shows that the procedure can restore urinary outflow but requires long-term follow-up.

## 1. Introduction

A urethrorectal fistula is an abnormal communication between the urethra and rectum that permits urine to enter the intestinal tract and may allow fecal material to contaminate the urinary tract. Reported clinical signs include passage of urine through the anus, watery or urine-contaminated feces, perineal wetness, recurrent urinary tract infection, and abnormal urination [1,2]. Acquired fistulas in small animals have been associated with pelvic trauma, surgery, prostatic disease, and local infection, whereas congenital fistulas are thought to result from abnormal partitioning of the embryonic cloaca [2,3].

Congenital urethrorectal communications are particularly rare in cats. Previously reported feline cases have involved anal atresia, disorders of sexual development, or broader urorectal septum malformations [4,5]. Diagnosis can be difficult because the fistulous tract may be narrow, anatomically deep, and accompanied by additional urinary or anorectal abnormalities. Positive-contrast studies, ultrasonography, CT, and endoscopy may therefore provide complementary information [1,6]. This report describes the diagnostic assessment and urinary diversion of a kitten with a patent anus, suspected distal urethral atresia, and a presumed congenital urethrorectal fistula. A modified perineal urethrostomy incorporating preputial mucosa was used to create a functional urinary outlet without direct fistula repair.

## 2. Case Description

### 2.1. Clinical Presentation and Laboratory Findings

A 4-week-old intact male domestic shorthair kitten weighing 745 g was presented after rescue from the street on 26 September 2023 (Table 1). The kitten had not been vaccinated and had received routine external parasite treatment. Appetite was initially normal, but multiple episodes of diarrhea developed the day after rescue. The feces became watery, malodorous, and intermittently blood-tinged. Enteritis had been suspected at another veterinary facility, but no specific treatment was administered. Persistent perianal wetness and apparent mixing of urine with feces prompted referral.

**Table 1:** Timeline of the diagnostic and therapeutic course.

Time Point	Clinical Findings and Diagnostic Assessment	Intervention and Outcome
26 September 2023	Kitten rescued; appetite initially normal.	Home observation.
Following day	Watery, malodorous diarrhea developed.	No specific treatment reported.
Referral presentation	BCS 3/9; 6% dehydrated; painful distended bladder; perianal wetness; anal fluid discharged during bladder compression.	Urinary-gastrointestinal communication suspected.
Diagnostic workup	Perianal fluid creatinine 1202 µmol/L with inflammatory cytology; ultrasound showed bladder-wall thickening and proximal urethral dilation; CT showed distal urethral discontinuity and a blind pelvic urethral pouch.	Presumed congenital urethrorectal fistula with suspected distal urethral atresia or discontinuity.
Surgery	Distal urinary outflow was nonfunctional; the suspected tract was not localized.	Modified perineal urethrostomy using preputial mucosa; suspected tract not explored or closed.
Postoperative day 3	Urine passed through the stoma; feces formed; mild leakage and minor incisional discharge persisted.	Catheter removed; wound care, analgesia, and antimicrobials continued.
Postoperative day 14	Clinically well; gaining weight; patent stoma; normal defecation; frequent intermittent leakage.	Skin sutures removed; no leakage-specific investigation.
Postoperative day 25	Incision dry; ultrasound showed no upper urinary tract dilation.	Discharged on day 25; long-term urinary surveillance advised.
Postoperative day 675	Stomal stenosis prevented normal urination.	A second surgery was performed.
Postoperative day 995	Urination generally unobstructed; intermittent leakage and hematuria persisted, although leakage frequency decreased and urinary control improved.	No leakage-specific investigation; no external stomal abnormality reported.

At presentation, body condition score was 3/9, rectal temperature was 38.9 °C, heart rate was 200 bpm, and respiratory rate was 44/min. Mucous membranes were pale pink, and dehydration was estimated at 6%. The urinary bladder was markedly distended and painful on abdominal palpation. Perianal hair was continuously wet, and dark green, watery, mucoid, malodorous fecal material was present. Gentle manual compression of the bladder consistently produced dark fluid from the anus, strongly suggesting an abnormal communication between the urinary and gastrointestinal tracts.

PCR assays on fecal material were negative for feline panleukopenia virus and feline coronavirus. Creatinine concentration in a sample of the dark, turbid perianal discharge was 1202 µmol/L. The high concentration was considered consistent with substantial urine contamination. Direct microscopy of the discharge identified numerous leukocytes, short rod-shaped bacteria, bacterial phagocytosis, and struvite crystals. Because the sample was collected from the perianal region rather than by cystocentesis, these findings were interpreted as evidence of local inflammation and mixed bacterial contamination rather than definitive bacterial cystitis.

Cystocentesis was not performed because safe urine collection in this small, uncooperative kitten would have required sedation, which the owner declined. Consequently, a sterile urine sample was not obtained, and urine culture and antimicrobial susceptibility testing were not performed.

No major abnormalities were identified on complete blood count or routine serum biochemistry. Potassium was 5.0 mmol/L (reference interval, 2.4–4.2 mmol/L), blood urea nitrogen was 35 mg/dL (15–34 mg/dL), extracellular fluid base excess was −8 mmol/L (−5 to 2 mmol/L), and serum amyloid A was 14.27 mg/L (0–2 mg/L). The modest increase in blood urea nitrogen and negative base excess were considered compatible with dehydration and a mild metabolic acid–base disturbance, whereas the increased serum amyloid A concentration supported systemic inflammation.

### 2.2. Imaging Findings and Diagnostic Assessment

Abdominal ultrasonography showed marked urinary bladder distension, diffuse bladder-wall thickening, and abundant echogenic suspended and dependent material within the lumen (Figure 1A). The proximal urethra was dilated to approximately 3.8 mm (Figure 1B). The left and right kidneys measured 36.1 and 36.7 mm in long-axis length, respectively, and the adjacent retroperitoneal soft tissues were hyperechoic (Figure 1C,D). No renal pelvic or ureteral dilation was detected. These findings supported impaired distal urinary outflow.

CT of the caudal abdomen and pelvis was subsequently performed in conjunction with retrograde positive-contrast urethrography. Contrast medium filled the penile urethra but did not demonstrate normal continuity with the remaining urethra. The pelvic urethra was dilated to approximately 4 mm. Dorsal to the caudal pubic margin, this structure expanded into a blind, diverticulum-like pouch measuring approximately 2.1 × 1.4 cm (Figure 2A,B). The urinary bladder was severely distended, and a 1.8 mm mineral-attenuating focus was present near the bladder neck (Figure 2C). The terminal left ureter appeared abnormally positioned near its vesical insertion, raising but not confirming the possibility of ectopic insertion (Figure 2D). Contrast passage into the rectum and the precise fistulous tract were not directly demonstrated.

The reproducible passage of fluid from the anus during bladder compression, the high creatinine concentration of that fluid, and the severe urethral abnormalities supported a presumptive diagnosis of congenital urethrorectal fistula with distal urethral atresia or discontinuity, proximal urethral dilation, and a blind pouch. Vesicorectal fistula, ureterorectal ectopia, and a broader urorectal septum malformation remained differential diagnoses because the communication was not visualized. The kitten’s age and absence of trauma or prior pelvic surgery made a congenital origin most likely.

The normal urinary pathway was nonfunctional. Retention of urine in the dilated proximal urethra and exposure to rectal contents increased the risks of ascending infection and urinary tract injury. Direct dissection of the tract was technically hazardous in a 745 g kitten because it could not be localized and the pelvic working space was extremely limited. The initial objective was therefore to establish a low-resistance urinary outlet and bypass the communication.

### 2.3. Therapeutic Intervention

The kitten was classified as American Society of Anesthesiologists physical status II and underwent general anesthesia. Positioned in dorsal recumbency, the perineal region and external genitalia were clipped, aseptically prepared, and draped. A modified perineal urethrostomy using preputial mucosa was performed as an adaptation of previously described techniques [7,8].

A circumferential incision was made at the cranial margin of the scrotum. The preputial mucosa was bluntly separated from the surrounding penile tissues while preserving a distal mucosal cuff (Figure 3A). The penis was mobilized by separating the ischiocavernosus muscles from the ischial arch, transecting the paired ischiourethral muscles, and dividing the retractor penis muscle (Figure 3B). The urethra was exposed and opened proximally toward the level of the bulbourethral glands. The distal penis was ligated and amputated.

A 3-Fr double-lumen urinary catheter was introduced through the preserved preputial opening and directed into the proximal urethral segment (Figure 3C). Guided by the preoperative imaging findings, the urethra was dissected proximally until the dilated segment and pouch-like region were entered and urine was released. Approximately 1 cm of additional mobilization was performed to obtain a patent lumen and permit tension-free mucosal apposition. After the mucosal margins were trimmed, the preputial mucosa was anastomosed to the urethral mucosa with 3-0 polyglycolic acid suture in a continuous pattern, creating a new epithelialized urethral stoma (Figure 3D). Catheter patency and the absence of gross tension were confirmed before routine closure of the surrounding soft tissues and skin. The suspected fistulous tract was not explored or closed.

### 2.4. Follow-Up and Outcomes

An Elizabethan collar was used to prevent self-trauma. Postoperative medications recorded in the clinical file included a single subcutaneous dose of meloxicam (0.2 mg/kg), intravenous amoxicillin-clavulanate (13.75 mg/kg), and a butorphanol constant-rate infusion (0.1 mg/kg/h) for 3 days. On postoperative day 3, the kitten was bright, eating, and passing urine through the stoma; fecal consistency had returned to normal. Mild urinary leakage persisted. A small amount of pale yellow purulent discharge was present at the incision margin, and the urinary catheter was removed. The site was cleaned, and treatment was continued with gabapentin (20 mg/kg orally every 12 h for 7 days). Because the kitten strongly resisted restraint and the owner declined cystocentesis, the presence of bacterial cystitis could not be confirmed. However, given the urinary–intestinal communication, the presence of cells containing phagocytosed bacteria in the urine-contaminated perianal discharge, and the purulent incisional discharge, empirical treatment with amoxicillin-clavulanate was administered to address the risk of ascending infection (12.5 mg/kg orally every 12 h for 30 days).

At postoperative day 14, the kitten remained clinically well, body weight was increasing, urine passed freely through the stoma, and defecation was normal. The skin sutures were removed. Frequent intermittent leakage from the stoma was reported, but no leakage-specific diagnostic investigation was performed; the stoma persisted, although the stoma remained patent and appeared externally normal. By postoperative day 25, the incision was dry and free of visible discharge, and the kitten was discharged from the hospital. An abdominal ultrasound examination performed at this visit showed a moderately filled bladder containing anechoic urine, with no evident intraluminal mass or mineralization. Both kidneys had normal contours and corticomedullary distinction, and no renal pelvic or ureteral dilation was identified.

Follow-up continued through postoperative day 995. At day 675, stomal stenosis prevented normal urination and necessitated a second surgery. At day 995, urination was generally unobstructed, and intermittent stomal leakage was less frequent with improved urinary control. Intermittent leakage and hematuria persisted, and no external abnormality of the stoma was reported.

## 3. Discussion

This case was most consistent with a presumed congenital urethrorectal fistula accompanied by suspected severe distal urethral maldevelopment. Congenital caudal urogenital and anorectal anomalies arise from abnormal partitioning of the embryonic cloaca, and the resulting phenotype depends on the extent and location of failed separation [3]. Feline reports are scarce and have generally involved anal atresia, abnormal sexual development, or a broader urorectal septum malformation sequence [4,5]. Unlike those reports, the present kitten had a patent anus and presented principally with apparent urinary discharge through the rectum and distal urinary outflow failure. This distinction is clinically relevant because the initial diagnostic focus can be incorrectly directed toward enteritis when watery feces and perianal wetness dominate the presentation.

The diagnosis should nevertheless be described as presumed rather than definitive. The high creatinine concentration in the perianal discharge and reproducible anal outflow during bladder compression provided strong evidence of urinary–intestinal communication. However, the suspected fistulous tract was not directly demonstrated by contrast imaging or surgery. A narrow tract, preferential flow from the urinary tract to the rectum, insufficient retrograde pressure, or segmental urethral obstruction could all have limited contrast opacification. Previous reports have used CT combined with colorectal dye infusion or positive-contrast urethrography to localize these communications [1,6]. Antegrade cystourethrography might improve opacification from the proximal side of the distal obstruction, whereas positive-contrast colonography or colorectal dye infusion could help identify an intestinal opening. CT fistulography could clarify the three-dimensional relationship of a tract to adjacent pelvic structures, and cystoscopy or proctoscopy could permit direct visualization and catheterization. The feasibility of these procedures would depend on patient size, clinical stability, and safe instrumentation.

The imaging findings also require cautious interpretation. The pouch-like structure was described as a urethral diverticulum in the original clinical assessment, but it may instead have represented marked dilation of a blind-ending pelvic urethral segment proximal to suspected congenital atresia. A vesicorectal fistula was considered less likely because the marked urethral discontinuity and blind pelvic urethral dilation favored a urethral origin, although bladder compression alone could not distinguish these possibilities. Isolated ureterorectal ectopia was less favored because anal discharge was reproducibly induced by bladder compression and no renal pelvic or ureteral dilation was identified; nevertheless, the abnormal terminal course of the left ureter was not investigated further, so ectopic insertion could not be excluded. A broader urorectal septum malformation was less typical because the anus was patent and the available imaging did not identify a more extensive composite anomaly, but this possibility also remained unresolved. Overall, the combination of a distended bladder, pressure-induced anal urine flow, and severe urethral discontinuity made a presumed urethral communication the leading diagnosis rather than a definitive anatomical conclusion.

Surgical management of urethrorectal fistula is usually directed toward separating and closing the urinary and rectal defects while preserving both lumens [2,9]. In the present kitten, direct repair was not selected because the suspected tract was not localized, the pelvic tissues were extremely small and fragile, and closing the suspected communication without restoring urinary outflow would not have resolved the suspected distal atresia. The modified perineal urethrostomy instead provided urinary diversion. The use of preputial mucosa creates a mucosa-to-mucosa junction and has been described as an alternative to conventional cutaneous urethrostomy in cats [7,8]. A recent retrospective evaluation of this technique in 30 cats reported generally favorable short-term outcomes, although wound dehiscence and stomal stricture remained possible [8]. Thus, the clinical contribution of the present report is the application of this established technique as urinary diversion for an unusual suspected congenital anomaly, rather than novelty of the technique itself. The procedure initially redirected most urine away from the rectum and restored formed feces, but it did not anatomically eliminate the suspected communication. Stomal stenosis subsequently prevented normal urination at day 675 and required a second surgery, demonstrating that favorable short-term urinary outflow did not ensure long-term stomal patency in this growing kitten.

Amoxicillin-clavulanate was continued for 30 days because the urinary–intestinal communication, bacterial phagocytosis in the urine-contaminated perianal discharge, and purulent incisional discharge raised concern for ascending infection. A sterile urine sample was not obtained, so treatment was empirical; culture-guided drug selection and duration would have been preferable.

Intermittent urinary leakage persisted from the early postoperative period through day 995, although its frequency decreased after the second surgery and urinary control improved. The leakage may have reflected incomplete continence at the stoma, congenital sphincter or innervation abnormalities, residual flow through the suspected communication, or dysfunction after prolonged distension of the bladder and proximal urethra. No diagnostic investigation was performed to determine the source of the leakage, and the cause of the hematuria reported at day 995 was not established. Long-term surveillance is important after feline urethrostomy because complications can include stomal stenosis, recurrent urinary tract infection, ascending urinary tract disease, and urinary incontinence [8,10]. The stenosis observed at day 675 illustrates the need for monitoring during growth because scar contraction, relative narrowing as the kitten matures, or both may compromise urinary outflow. Serial assessment should include inspection of the stoma, urinalysis and urine culture when indicated, renal biochemical markers, and ultrasonographic evaluation of the kidneys and ureters.

This report has several limitations. The suspected fistulous tract and its rectal opening were not directly visualized or surgically explored; therefore, the exact anatomical diagnosis remains uncertain. The suspected ectopic left ureter was not investigated further. Urine culture and antimicrobial susceptibility testing were not performed, and the prolonged antimicrobial treatment was empirical. Although follow-up extended to day 995 and documented stomal stenosis requiring a second surgery, the persistent leakage and hematuria were not objectively investigated, limiting assessment of long-term urinary function.

## 4. Conclusions

Congenital urinary–rectal communication should be considered in kittens with unexplained watery feces, persistent perianal wetness, or fluid discharged from the anus during bladder compression. Fluid creatinine measurement and multimodal imaging can support a presumptive diagnosis, although direct delineation of the tract is required for definitive anatomical classification. When suspected distal urethral atresia prevents normal urinary outflow and direct repair of the suspected communication is impractical, a modified perineal urethrostomy using preputial mucosa may provide effective urinary diversion. In this kitten, later stomal stenosis required a second surgery, and intermittent leakage and hematuria persisted at day 995; continued monitoring is therefore essential throughout growth and thereafter.

## Figures and Tables

**Figure 1:**
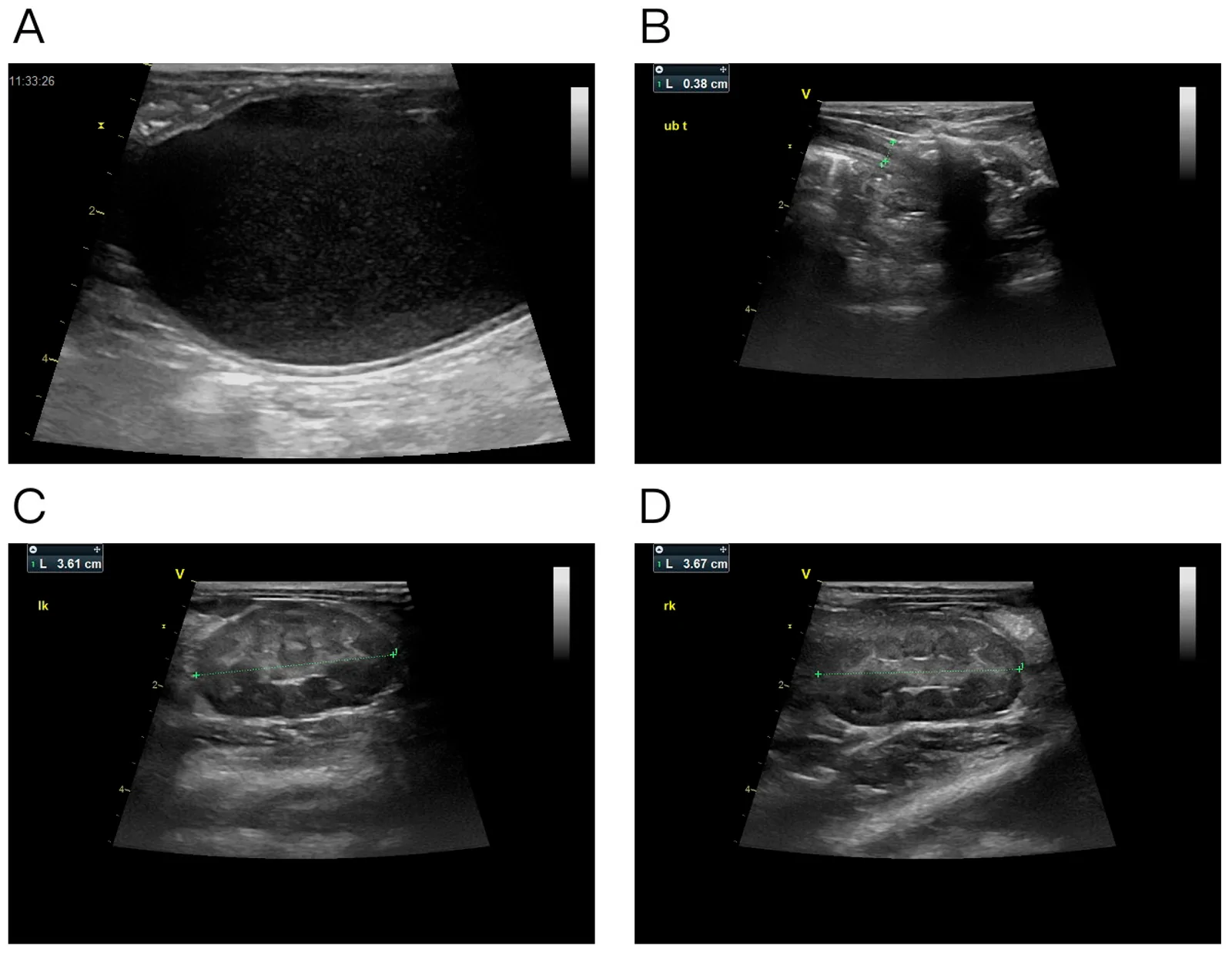
Abdominal ultrasonographic findings. (**A**) Markedly distended urinary bladder containing diffuse suspended echoes and dependent echogenic material. (**B**) Dilated proximal urethra measuring approximately 3.8 mm. (**C**,**D**) Long-axis images of the left and right kidneys, measuring 36.1 and 36.7 mm, respectively.

**Figure 2:**
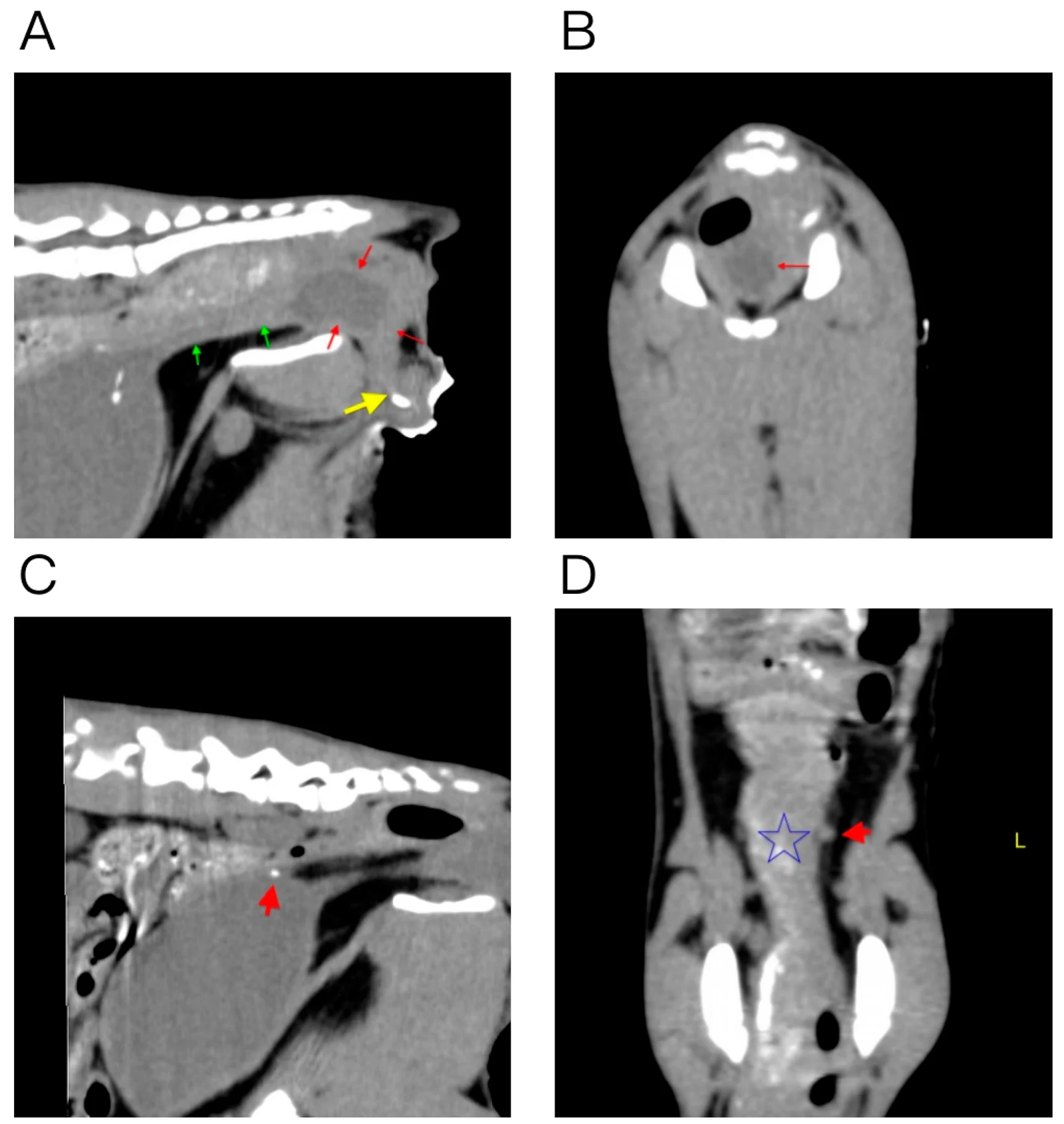
CT and retrograde urethrographic findings. (**A**) Sagittal image of the caudal pelvis. Green arrows indicate the dilated pelvic urethra, red arrows indicate the blind diverticulum-like expansion, and the yellow arrow indicates contrast medium within the penile urethra. (**B**) Transverse image at the level of the pouch-like urethral dilation (red arrow). (**C**) Sagittal image of the urinary bladder showing a small mineral-attenuating focus near the bladder neck (red arrow). (**D**) Coronal image of the caudal abdomen and pelvis. The star marks the rectum, and the red arrow indicates the terminal left ureter.

**Figure 3:**
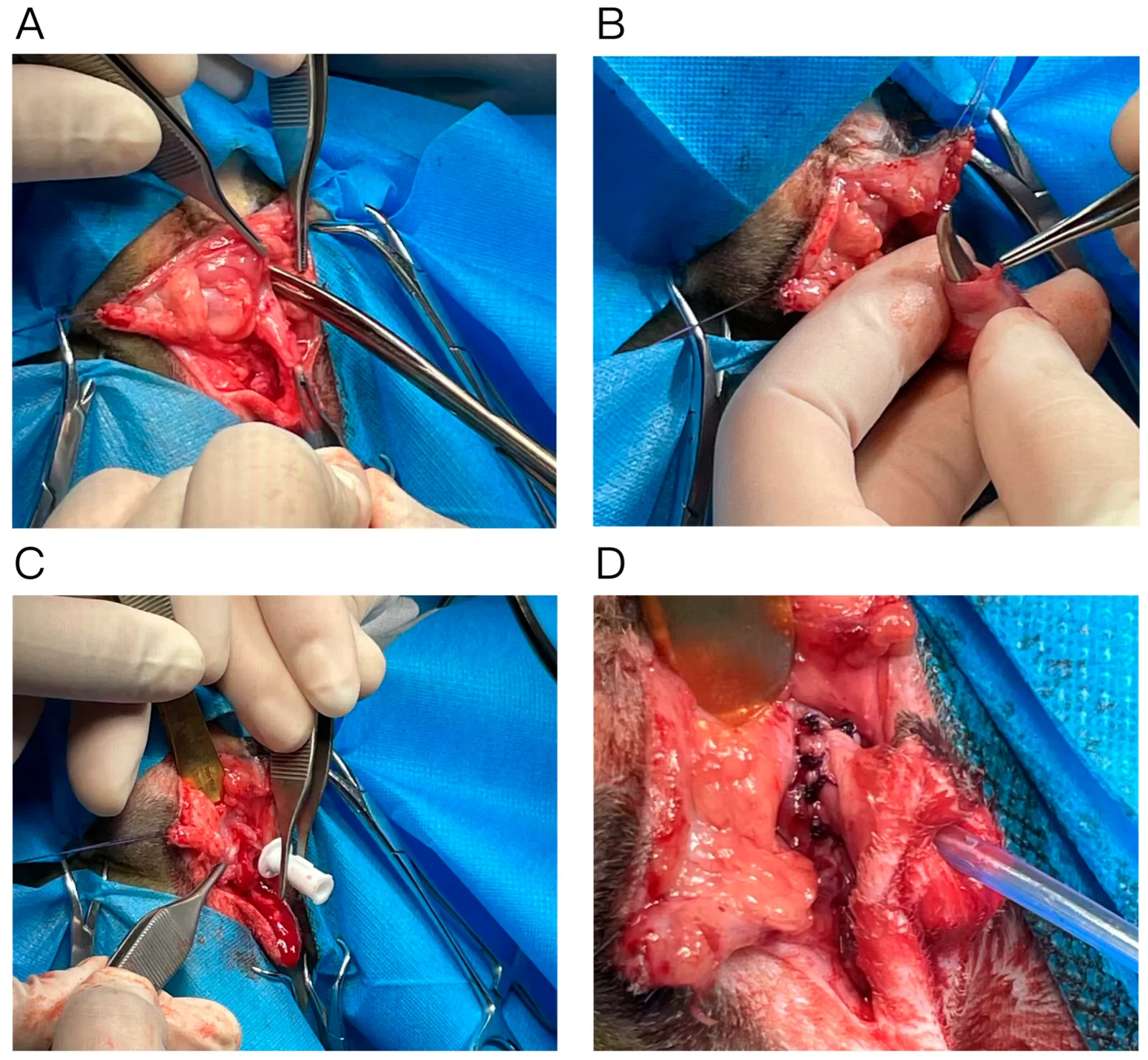
Intraoperative photographs of the modified perineal urethrostomy. (**A**) Separation and preservation of the preputial mucosa. (**B**) Mobilization of the penis and associated soft tissues. (**C**) Placement of a 3-Fr urinary catheter through the preserved preputial opening after distal penectomy. (**D**) Mucosa-to-mucosa anastomosis between the proximal urethra and preputial mucosa, creating the new urethral stoma.

## Data Availability

The original contributions presented in the study are included in the article. Further inquiries can be directed to the corresponding author.
